# *Leishmania donovani*-Induced Increase in Macrophage Bcl-2 Favors Parasite Survival

**DOI:** 10.3389/fimmu.2016.00456

**Published:** 2016-10-25

**Authors:** Rajeev Kumar Pandey, Sanjana Mehrotra, Smriti Sharma, Ramachandra Subbaraya Gudde, Shyam Sundar, Chandrima Shaha

**Affiliations:** ^1^Cell Death and Differentiation Research Laboratory, National Institute of Immunology, New Delhi, India; ^2^Department of Human Genetics, Guru Nanak Dev University, Amritsar, India; ^3^Department of Medicine, Institute of Medical Sciences, Infectious Disease Research Laboratory, Banaras Hindu University, Varanasi, India; ^4^Central Animal Facility, Indian Institute of Science, Bangalore, India

**Keywords:** *Leishmania donovani*, Bcl-2, macrophage, nitric oxide, IL-13, ABT-199

## Abstract

Members of the Bcl-2 family are major regulators of apoptosis in mammalian cells, and hence infection-induced perturbations in their expression could result into elimination of the parasites or creation of a niche favoring survival. In this investigation, we uncover a novel role of host Bcl-2 in sustaining *Leishmania donovani* infection. A rapid twofold increase in Bcl-2 expression occurred in response to parasite challenge. Downregulation of post infection Bcl-2 increase using siRNA or functional inhibition using Bcl-2 small molecule inhibitors interfered with intracellular parasite survival confirming the necessity of elevated Bcl-2 during infection. An increased nitric oxide (NO) response and reduced parasitic burden was observed upon Bcl-2 inhibition, where restitution of the NO response accounted for parasite mortality. Mechanistic insights revealed a major role of elevated Th_2_ cytokine IL-13 in parasite-induced Bcl-2 expression *via* the transcription factor STAT-3, where blocking at the level of IL-13 receptor or downstream kinase JAK-2 dampened Bcl-2 induction. Increase in Bcl-2 was orchestrated through Toll like receptor (TLR)-2-MEK-ERK signaling, and changes in TLR-2 levels affected parasite uptake. In a mouse model of visceral leishmaniasis (VL), Bcl-2 inhibitors partially restored the antimicrobial NO response by at least a twofold increase that resulted in significantly reduced parasite burden. Interestingly, monocytes derived from the peripheral blood of six out of nine human VL subjects demonstrated Bcl-2 expression at significantly higher levels, and sera from these patients showed only marginally quantifiable nitrites. Collectively, our study for the first time reveals a pro-parasitic role of host Bcl-2 and the capacity of host-derived IL-13 to modulate NO levels during infection *via* Bcl-2. Here, we propose Bcl-2 inhibition as a possible therapeutic intervention for VL.

## Introduction

Pathogens manipulate host-cell proteins to survive, and the ones supporting their persistence form attractive targets for development of drugs. Apoptosis is a cellular process, frequently encountered to be manipulated during infection (1). During the course of a pathogenic invasion, host-cell apoptosis serves as an innate immune mechanism by warding off the multiplication and the spread of the pathogen at the cost of the infected cell (2, 3). The pathogen on the other hand antagonizes this defense by manipulating host-cell proteins regulating this very process (1, 4). The Bcl-2 family of pro and antiapoptotic regulatory proteins expressing Bcl-2 homology or BH domains primarily controls the well-orchestrated process of apoptosis within mammalian cells along with other related functions (5). These proteins are druggable targets because of their involvement in vital cellular functions. Interestingly, this family also represents one such class of molecules that is modulated during bacterial and viral infections (1, 6, 7), however, their role in protozoal infection is not known. Bcl-2 family proteins expressing four BH domains are antiapoptotic, like Bcl-2, Mcl-1, and Bcl-xL, and pro-apoptotic proteins like Bad and Bik express three or one BH domains respectively (8). The different members of the family form homo or heterodimers through the BH3 domain to regulate various cellular functions including autophagy and apoptosis. Understanding of this binding has led to the development of BH3 mimetics capable of interfering with functions of proteins like Bcl-2, Bcl-xL, and Mcl-1. ABT-263 is a small molecule inhibitor for Bcl-2, Bcl-xL, and Bcl-w while ABT-199 is a specific inhibitor for Bcl-2, which is in clinical trials for cancer treatment (9, 10). While ABT-263 is still in phase-II trials, ABT-199, created by re-engineering of ABT-263 to make it more Bcl-2-specific, has been given the “Breakthrough Therapy Designation” by the FDA in 2015 for the treatment of lymphoid malignancies.

The involvement of the Bcl-2 family of proteins in preventing or inducing host-cell death has been explored for bacterial pathogens like *Mycobacterium tuberculosis* where infection upregulates Bcl-2 in mouse macrophages and THP-1 cells through the mitogen-activated protein kinase (MAPK) pathway (11). Another bacterial pathogen *M. leprae*, downregulates pro-apoptotic Bcl-2 family members Bad and Bak and upregulates the antiapoptotic Mcl-1 protein to prevent host-cell apoptosis (12). However, in all the above studies, functional involvement of Bcl-2 is not well elucidated.

While the mechanisms of *Leishmania* infection have been widely explored, there is no information on how host Bcl-2 behaves during infection with this parasitic protozoan. For elimination of the *Leishmania* parasite, the microbicidal molecule nitric oxide (NO) produced by the activity of the inducible nitric oxide synthase (iNOS) enzyme is crucial (13, 14). Deficient or impaired NO response leads to resistance to antimonial drugs, the main line of therapy against visceral leishmaniasis (VL) (15). The sensitivity of the *Leishmania* parasite to NO suggests that boosting of mechanisms that allow NO to be generated in infected cells would be an effective means of eliminating infection. Bcl-2 is known to suppress NO production in mammalian cells (16) but it is not known if Bcl-2 influences NO production in macrophages during *L. donovani* infection.

The idea that Bcl-2 family proteins play a role in pathogen survival prompted us to check if they were also important for the success of *L. donovani* infection and if interference with these proteins could abridge parasitic burden. Using both *in vitro* and *in vivo* experimental systems, we, demonstrate increased expression of the Bcl-2 protein in response to *L. donovani* infection and also show that suppression or functional inhibition of the infection-induced Bcl-2 results in accelerated clearance of the parasites. Our results assign a pro-parasitic role to the cytokine IL-13 by showing its involvement in the suppression of NO production *via* Bcl-2 induction. We also present evidence in support of the involvement of the host surface toll-like receptor (TLR)-2 in Bcl-2 induction and in the early interaction and subsequent internalization of the parasites by the macrophages. The data demonstrates a new possibility of use of Bcl-2 small molecule inhibitors as antileishmanial agents.

## Materials and Methods

### Ethics Statement

All animal experiments were duly approved by the Institutional Animal Ethics Committee of the National Institute of Immunology, New Delhi (IAEC/AQ/2015/134, serial no. IAEC#372/15). Use of human peripheral blood monocytes (PBMCs) was approved by the Institutional Human Ethics Committee of the National Institute of Immunology, New Delhi (Project no. IHEC #84/14). The work involving human subjects was also approved by the ethical committee of the Institute of Medical Science, Banaras Hindu University, Varanasi, India, and the informed consents were obtained from the patients or their guardians.

### Study Subjects

Subjects with symptoms of VL were recruited to Kala-Azar Medical Research Centre, Muzaffarpur, Bihar, India, where they were confirmed to be positive for VL by detection of amastigotes in the splenic aspirates and/or by rK39 positive test. All patients included in the study were HIV negative and over 6 years of age. Splenic aspiration is the gold standard method for diagnosis of VL; splenic aspirates were collected before the start of antileishmanial treatment and after cure for examining parasitological status of subjects. Subjects with low hemoglobin, platelet count less than 40,000/μl and prothrombin time <5 s were excluded from the study. Venous blood from VL patients and endemic controls (EC) was collected in heparin. All EC were healthy household members of the patients.

### Animals

For *in vivo* experiments, inbred BALB/c mice were used. For *in vitro* experiments, mouse peritoneal macrophages (mPMs) were obtained from inbred mice of BALB/c strain, wild-type, and TLR-2 knockout mice of C57/BL6 background of either sex at 6–10 weeks of age.

### Cells and Culture Conditions

THP-1 cells, an acute monocytic leukemia-derived human cell line (ATCC TIB-202TM) were maintained in RPMI-1640 (Sigma-Aldrich, Cat. No. R8005) medium supplemented with 10% heat-inactivated fetal bovine serum (FBS) (Gibco, Cat. No. 10082147) and penicillin (100 U/ml), streptomycin (100 U/ml), and gentamycin (20 μg/ml) at 37°C in humidified air containing 5% CO_2_. For differentiation into macrophages, they were incubated with 50 ng/ml phorbol 12-myristate 13-acetate (PMA) (Sigma-Aldrich, Cat. No. P1585) as described previously. Human PBMCs were isolated using a Ficoll-Paque density gradient (Sigma-Aldrich, Cat. No. GE-17-5442-02) from whole blood. Briefly, 2 × 10^6^ monocytes were incubated in the presence of recombinant human M-CSF (300 ng/ml) (Shenandoah Biotechnology Inc., Cat. No. 0114-100-03AF). Non-adherent cells were periodically removed followed by addition of fresh media containing recombinant human M-CSF (300 ng/ml) and 10% human serum every 12 h. Cells were allowed to differentiate into human monocyte derived macrophages (hMDMs) for 96 h.

Mouse peritoneal macrophage monolayers were prepared as described previously (17). Briefly, peritoneal exudate cells were harvested from BALB/c or C57/BL6 mice using chilled serum-free RPMI 1640 medium, incubated for 2 h, non-adherent cells were removed and the adherent cells were further incubated in complete RPMI 1640 medium to form macrophage monolayer.

The parasite, *L. donovani* (BHU1260), was derived from the primary culture obtained directly from the splenic aspirate of a human VL patient and was maintained in M-199 medium (Sigma-Aldrich, Cat. No. M5017) supplemented with 10% heat-inactivated FBS at 23°C as described previously. The primary cultures were used only up to fourth passage and exhibited good infectivity *in vitro*.

### Treatments

For siRNA transfection, THP-1 MDMs, or hMDMs were transfected with gene-specific siRNAs (Bcl-2, Mcl-1, TLR-2, IL-13R) or scrambled negative control siRNA at a final concentration of 25 nM. Transfection was performed using N-TER™ Nanoparticle siRNA Transfection System as per manufacturer’s instructions (Sigma Aldrich, Cat. No. N2913). Plasmid containing Bcl-2 clone was transfected using Lipofectamine LTX reagent with PLUS reagent (Thermo Fisher Scientific, Cat. No. 15338100). During the entire course of study, the Bcl-2 selective small molecule inhibitor ABT-199 (10 nM) and in selective experiments, another Bcl-2 inhibitor ABT-263 (10 nM) was used to inhibit Bcl-2 activity. For inhibition of specific cell signaling pathways, pharmacological small molecule inhibitors of various signaling molecules were added 30 min prior to the start of the experiments. The inhibitors used were: 1, 4-diamino-2,3-dicyano-1,4-bis (*o*-aminophenylmercapto) butadiene monoethanolate (U0126) (2 μM) (Sigma Aldrich, Cat. No. U120) and 2-(2-Amino-3-methoxyphenyl)-4H-1-benzopyran-4-one (PD98059) (10 μM) (Sigma Aldrich, Cat. No. P215) for MEK1/2; 2-(4-Morpholinyl)-8-phenyl-1(4H)-benzopyran-4-one hydrochloride (LY294002) (30 μM) (Sigma Aldrich, Cat. No. L9908) and wortmannin (100 nM) (Sigma Aldrich, Cat. No. W1628) for phosphatidylinositol 3-kinase (PI3-K); and Tyrphostin (AG490) (10 μM) (Calbiochem, Cat. No. 658401) for STAT-3.

### Determination of Intracellular Parasite Burden

Late stationary phase promastigotes were used to challenge macrophages at multiplicity of infection (MOI) of 10 parasites per macrophage. After 2 h of incubation, the unbound parasites were removed by gently shaking and washing the wells with incomplete RPMI-1640 medium three times and the infected macrophage monolayers were incubated in the complete medium for 12 or 24 h. To estimate the intracellular parasitic burden, cells were fixed in 4% paraformaldehyde (PFA) followed by staining with the DNA fluorescent probe DRAQ5 (5 μM) (Thermo Fisher, Cat. No. 62251) for 10 min. After washing three times with sterile PBS to remove the excess stain, cells were mounted in Vectashield anti-fade mounting medium (Vector Laboratories, Inc., Burlingame, CA, USA) and were used to acquire images using Leica TCS SP5 II (Leica Microsystems, Wetzlar, Germany) confocal microscope at 633 nm. Infecting the cells at the mentioned MOI resulted in a situation where 98–99% cells were infected with at least one parasite. At least 200 cells observed from a minimum of 15 randomly selected fields were counted manually for each condition to determine the average number of parasites per macrophage. Similarly, for estimating parasite uptake, a 2-h infection period was used after which cells were processed, stained with DRAQ5, and average parasite counts per macrophage were made. The parasite counting was performed by a person in the laboratory blinded for the experimental conditions, other than the person performing the experiment.

### Immunohistochemistry

Cells fixed with 2% PFA were permeabilized with 0.1% Triton X-100 and incubated overnight with primary antibody against Bcl-2 (1:200) (Millipore, Cat. No. 05-729) or TLR-2 (1:250) (Novus Biologicals, Cat. No. NBP1-77843) at 4°C. Secondary antibody conjugated to Alexa 488 (1:400) (Invitrogen, Cat. No. A11001) was used to detect staining at excitation/emission wavelength: 490/525 nm. DNA staining was performed using DAPI (10 μg/ml) (excitation/emission wavelength: 647/681 nm) (Thermo Fisher, Cat. No. 62251). Images were captured using Leica TCS SP5 II (Leica Microsystems, Wetzlar, Germany) confocal microscope and analyzed using an integrated Leica LAS AF software program.

### Protein Extraction and Western Blots

Cells were lysed in cell lysis buffer as described earlier (18). Cell lysates (30–80 μg) electrophoresed on 12% SDS polyacrylamide gels were transferred onto nitrocellulose membranes. Membrane blocking was performed in 5% fat free milk (Santa Cruz Biotechnology, Cat. No. sc-2325) followed by incubation with any of the primary antibodies like, Bcl-2 (1:1000) (BD Biosciences, Cat. No. 51-6511GR), Bcl-xL (1:1000) (Cell Signaling Technologies, Cat. No. 54H6), Mcl-1 (1:1000) (BD Biosciences, Cat. No. 559027), Bad (1:1000) (Santa Cruz Biotechnology, Cat. No. sc-492), p-Bad (1:1000) (Santa Cruz Biotechnology, Cat. No. sc-271963), TLR-2 (1:1000) (Novus Biologicals, Cat. No. NBP1-77843), pERK-1/2 (1:1000) (Cell Signaling Technologies, Cat. No. 9101), p-STAT-3 (1:1000) (Cell Signaling Technologies, Cat. No. D3A7), p-PI3K (1:1000) (Cell Signaling Technologies, Cat. No. 4428), p-Akt (1:1000) (Cell Signaling Technologies, Cat. No. 9271), iNOS (1:1000) (Santa Cruz Biotechnology, Cat. No. sc-8310), and β-tubulin (1:4000) (Thermo, Cat. No. RB-9249-P1). Subsequently, post wash, blots were incubated with corresponding secondary antibodies conjugated to horseradish peroxidase. Protein bands were observed on X-ray film using femtoLUCENT™ PLUS-HRP reagent (G-biosciences, Cat. No. 786-003). To monitor for equal loading of proteins in each well, Western blotting using antibody against β-tubulin was performed for each experiment.

### Measurement of Nitrite Production

The concentration of nitrite, the stable end product of NO, in culture supernatants or serum was determined by Griess’ reaction as described by Biswas et al. with slight modifications (19). Briefly, 10^6^ cells were plated in the wells of a 12-well culture plate, and after the completion of the experiments the cell-free supernatant from individual wells were collected and stored until use. About 100 μl of the sera or culture supernatants were incubated with equal volume of Griess’ reagent for 20 min at room temp. Spectrophotometric readings were taken at 540 nm in a microplate reader (μQuant, BioTek Instruments Inc., VT, USA). Nitrite content was quantified by extrapolation from a sodium nitrite standard curve for each experiment.

### Enzyme-Linked Immunosorbent Assay for IL-13

IL-13-specific enzyme-linked immunosorbent assay (ELISA) was performed to detect level of secreted IL-13 in the cell-free supernatant obtained from different *in vitro/ex vivo* experiments or serum of mice using human/mouse-specific IL-13 ELISA kits (e-Biosciences, Cat. Nos. 88-7439-22, human; 88-7137-22, mouse) as per manufacturer’s instructions.

### *In Vivo* Infection Experiments

Inbred BALB/c mice were grouped as untreated non-infected control, infected, infected vehicle control, and infected drug treated. Mice in last three groups were inoculated with *Leishmania* promastigotes through intra-cardiac injection with 50 μl bolus containing 1 × 10^7^ parasites on day 0 of the study. Drug (ABT-199, ABT-263 both at 15 mg/kg body weight) or vehicle (DMSO) was injected through tail vein. In the drug or vehicle control groups, injections were given for first 3 weeks, once every week. Mice were humanely euthanized after 5 weeks and spleens were dissected out, weighed, and impression smears were made. Parasite burden was estimated by counting splenic smears prepared after euthanasia. Blood was collected from mice and plasma was extracted for detection of nitrite and IL-13 levels in circulation.

### Statistical Analysis

Standard one-way analysis of variance (ANOVA) followed by Bonferroni’s multiple comparison test was performed to derive the statistical significance between the test groups while comparing densitometric data of Western blots. For comparing nitrite concentration, parasitic burden, and mice data between different test groups, where information about a Gaussian distribution could not be conclusively derived, Mann–Whitney test was performed. Wilcoxon signed-rank test was applied to analyze the data from the paired samples. The statistical analysis was performed using GraphPad Prism, version 5.01 (GraphPad, San Diego, CA, USA). *P*-value of less than 0.05 was considered significant. The error bars of the values represent ± SEM from the replicates.

## Results

### Expression of Bcl-2 Family Proteins Changes in Response to *Leishmania* Infection

**Figure 1:**
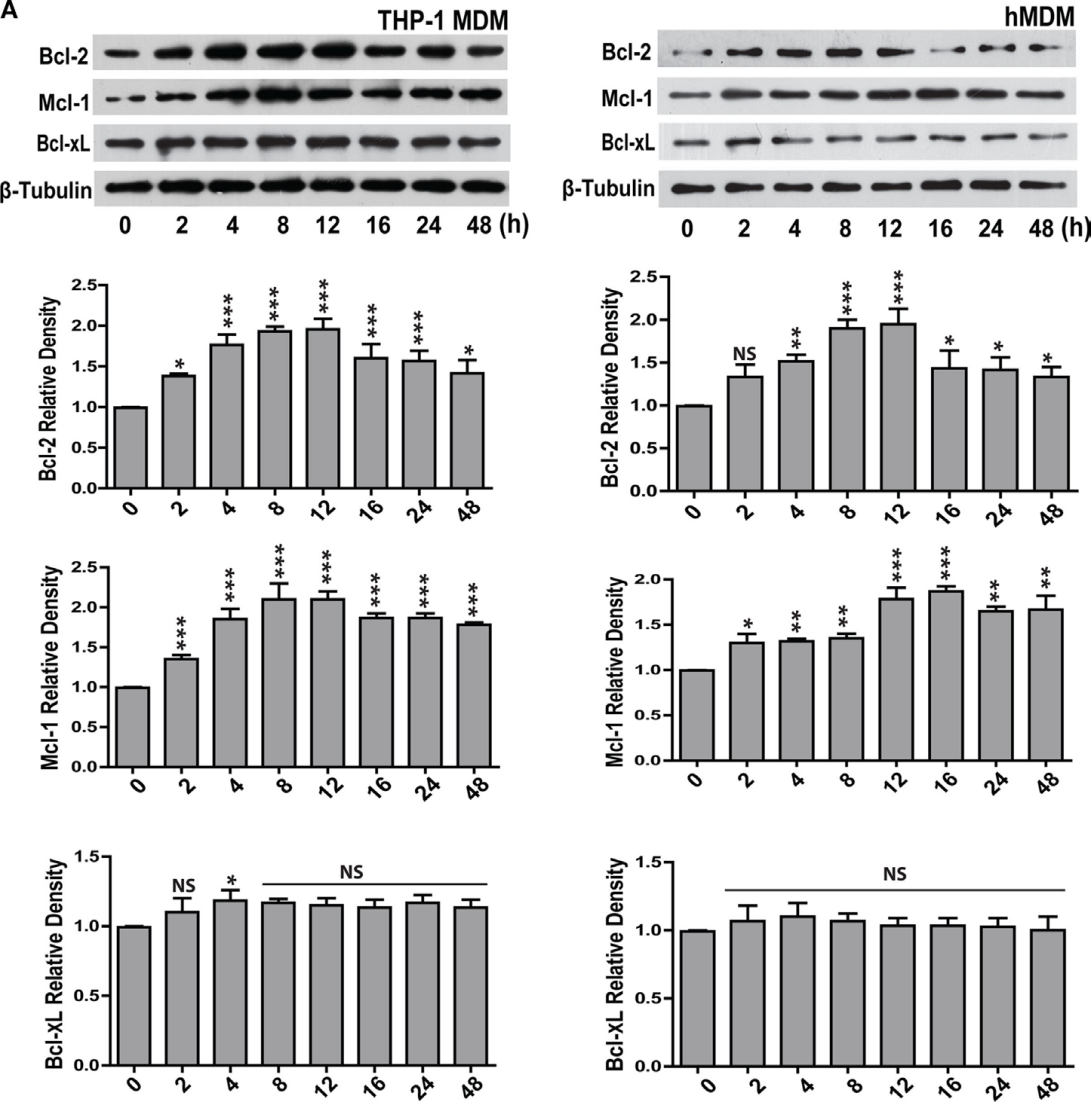

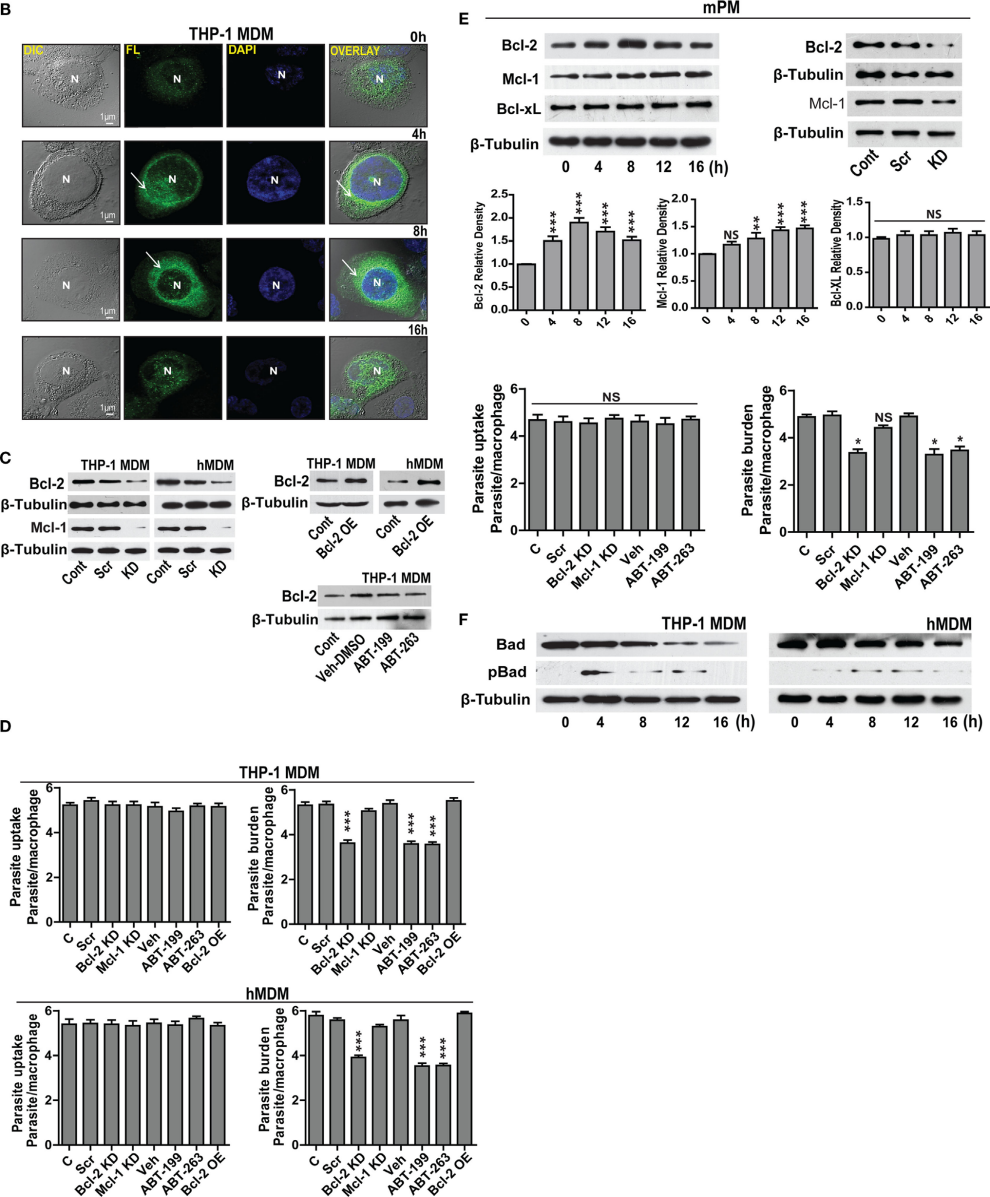
**Expression of Bcl-2 and Mcl-1 increases during *L. donovani* infection**. **(A)** Bcl-2, Mcl-1, and Bcl-xL protein levels in extracts of THP-1 MDM and hMDM infected with *L. donovani* parasites *in vitro*. Note the increase in Bcl-2 from 2 h onward. Blots are representative of three experiments. Densitometric plots are shown below. Data are mean ± SD (*n* = 3). **(B)** Immunocytochemical staining of infected THP-1 MDM using anti-Bcl-2 antibody at different time points post-infection. Note increased Bcl-2 expression at 8 h showing predominantly perinuclear distribution as indicated by white arrows. N = nucleus. **(C)** Blots showing siRNA mediated downregulation of Bcl-2 and Mcl-1 in THP-1 MDM and hMDM. Control is without any siRNA. Scr, scrambled siRNA; KD, knockdown. Also shown is overexpression of Bcl-2 in THP-1 MDM and hMDM. OE, overexpression. Control is only plasmid, OE, overexpression. Blots are representative of three experiments. The blot below shows control Bcl-2 levels during treatment with only vehicle (DMSO) or ABT-199 or ABT-263. Bcl-2 levels remained unaffected upon these treatments. **(D)** Plots show parasite uptake and parasite burden during various conditions. Average parasite counts per macrophage have been put on the *y*-axis. A minimum of 200 cells was counted. Data are mean ± SEM (*n* = 3); ***P ≤ 0.001; Mann–Whitney test. Parasite uptake remained unaltered. Note significant reduction of parasite burden in siRNA or ABT-199-treated Bcl-2 downregulated cells. **(E)** Western blots of lysates of infected mPM stained for Bcl-xL, Bcl-2, and Mcl-1 showing increased expression for Bcl-2 which peaks at 8 h post-infection. Blots are representative of three experiments. Shown below are the densitometric plots. Data are mean ± SD (*n* = 3). Alongside are blots showing siRNA0-mediated downregulation of Bcl-2 and Mcl-1 in mPMs. Control is without any siRNA. Scr, scrambled siRNA; KD, knockdown. Shown below are bar graphs expressing parasite uptake and parasite burden during various treatments. Note significantly reduced parasite burden in Bcl-2 KD and ABT-199/ABT-263-treated macrophages. A minimum of 200 cells was counted. Data are mean ± SEM (*n* = 3); *P ≤ 0.05; Mann–Whitney test. **(F)** Western blots from THP-1 MDM and hMDM showing time-dependent degradation of Bad protein during infection. Bad protein undergoes degradation post phosphorylation. The blot below shows Bad phosphorylation upon infection. Blots are representative of three experiments.

For investigating the changes in the expression of Bcl-2 family proteins after infection with *L. donovani* parasite, THP-1 monocyte-derived macrophages (THP-1 MDM) were challenged at MOI of 1 macrophage:10 parasites (Figure S1A in Supplementary Material) *in vitro*, and the levels of Bcl-2 family proteins were measured. A time-dependent increase in Bcl-2 was observed post infection (Figure 1A). The increase in Bcl-2 dominated from 2–12 h following which there was a gradual decline. Mcl-1 protein expression also increased following infection and the elevated levels were sustained. Bcl-xL levels did not change significantly during the course of infection (Figure 1A). Likewise, when primary human macrophages derived from blood monocytes, i.e., hMDMs were infected with the parasites (Figure S1A in Supplementary Material), similar changes were observed. Both Bcl-2 and Mcl-1 levels increased with almost identical pattern and kinetics as that of THP-1 MDMs (Figure 1A). An investigation into intracellular Bcl-2 immunostaining demonstrated predominantly perinuclear localization of the protein in THP-1 MDM (Figure 1B).

Concomitant increase in the levels of the antiapoptotic proteins Bcl-2 and Mcl-1 and decrease in the level of the pro-apoptotic protein Bad implied enhanced survival of the parasite-infected macrophages. Infected cells by default experience higher levels of cellular stress and therefore are at much higher risks of undergoing apoptosis. Increasing expression of antiapoptotic proteins or reducing expression of pro-apoptotic proteins of the host cells are among well-documented pro-pathogenic strategies and can be critically effective during establishment of infection (1). To explore if increased Bcl-2 and Mcl-1 served any pro-parasitic intent by imparting higher survival advantage to the infected cells, macrophages were knocked down for Bcl-2 or Mcl-1 (Figure 1C), and cell viability was checked at different time points post infection. Macrophages knocked down for either Bcl-2 or Mcl-1 showed no significant impact on viability upto 48 h of infection (Figure S1B in Supplementary Material). However, Bcl-2 knocked down macrophages showed increased cell death as compared with control cells at 72 h post-infection. Similar to Bcl-2 downregulated cells, Mcl-1 knocked down macrophages showed reduced viability at 72 h, however to a lesser extent (Figure S1B in Supplementary Material). To look for any potential impact on parasite internalization introduced due to knocking down of Bcl-2/Mcl-1, macrophages in different groups were infected for 2 h and average parasite numbers were counted. No significant differences in the average number of internalized parasites were found among different groups, negating any possible effect of Bcl-2/Mcl-1 knock down on parasite uptake (Figure 1D). To look for possible role of Bcl-2 and Mcl-1 in the sustenance of the parasite inside macrophages, parasite burden was estimated under Bcl-2/Mcl-1 knock-down conditions at 12 h of infection. Cells treated with Bcl-2 siRNA showed a pronounced reduction in the number of parasites/cell as compared to the scrambled siRNA control. However, unlike Bcl-2 knocked down cells, Mcl-1 knocked down macrophages did not show any significant reduction in parasite number (Figure 1D). To further look if this effect was specific to Bcl-2, Bcl-2 overexpression (Figure 1C) was carried out in Bcl-2 lowered cells before they were challenged with parasites. Bcl-2 overexpressing macrophages showed parasite burden comparable to control cells (Figure 1D). These observations suggested that elevated presence of Bcl-2 but not Mcl-1 favored parasite survival. The siRNA-mediated downregulation of Bcl-2 in hMDM also resulted in lesser infection rates as compared to scrambled siRNA-treated cells (Figure 1D). Consequently, to reaffirm the role of Bcl-2 and further establish its functional relevance, we used Bcl-2 inhibitors ABT-199 and ABT-263 to block Bcl-2 function through binding site interference. Parasite challenge during ABT-199 or ABT-263 mediated inhibition of Bcl-2 in both THP-1 MDM and hMDM showed lower infection as compared to vehicle-treated controls (Figure 1D, ABT-199, ABT-263). Having obtained positive correlation between Bcl-2 expression and parasite burden in human macrophages, we considered it relevant to check this hypothesis in an animal model. Before moving onto a mouse model, which is one of the two established animal models for VL, it was important to look at the status of Bcl-2 family proteins in mouse macrophages. Infection experiments with mPMs reflected similar alterations in the profile of Bcl-2 family proteins *in vitro*, albeit, with a slightly different time kinetics. Here, the Bcl-2 increase peaked at 8 h followed by a decline and Mcl-1 showed an increase from 8–16 h with Bcl-xL showing no noteworthy change (Figure 1E). Similar to human macrophages, mPMs with Bcl-2 knock down or in the presence of Bcl-2 functional inhibitors did not show reduced parasite uptake, but demonstrated lower parasite burden (Figure 1E). Among the pro-apoptotic members of the Bcl-2 family, only Bad protein showed significantly altered expression. There was a gradual decline in Bad levels pertaining to its rapid phosphorylation post infection (Figure 1F). Taken together, the above data clearly provided evidence for a supportive role of Bcl-2 to parasite survival within the host cells.

### Bcl-2 Levels Influence Generation of NO in Macrophages

**Figure 2:**
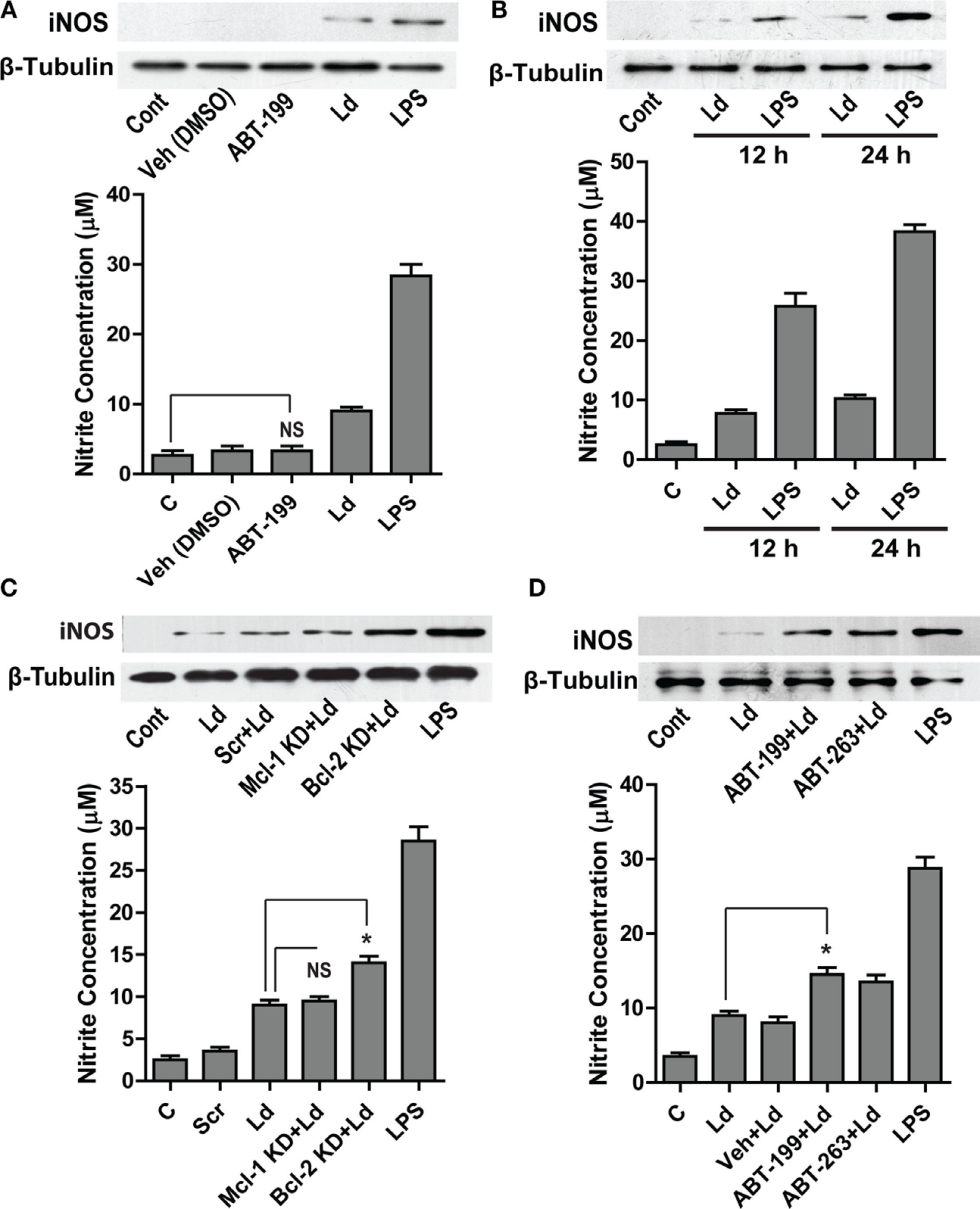

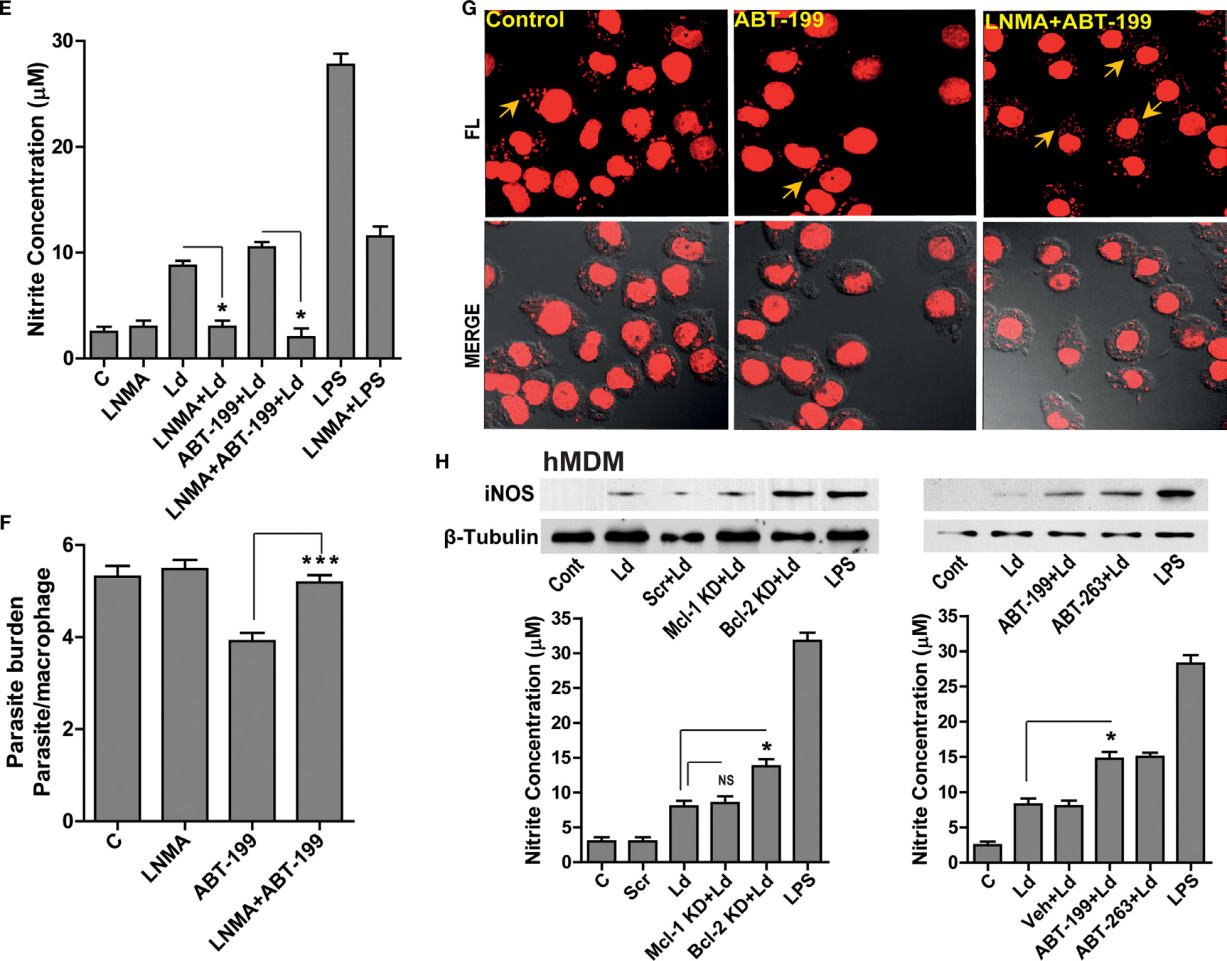

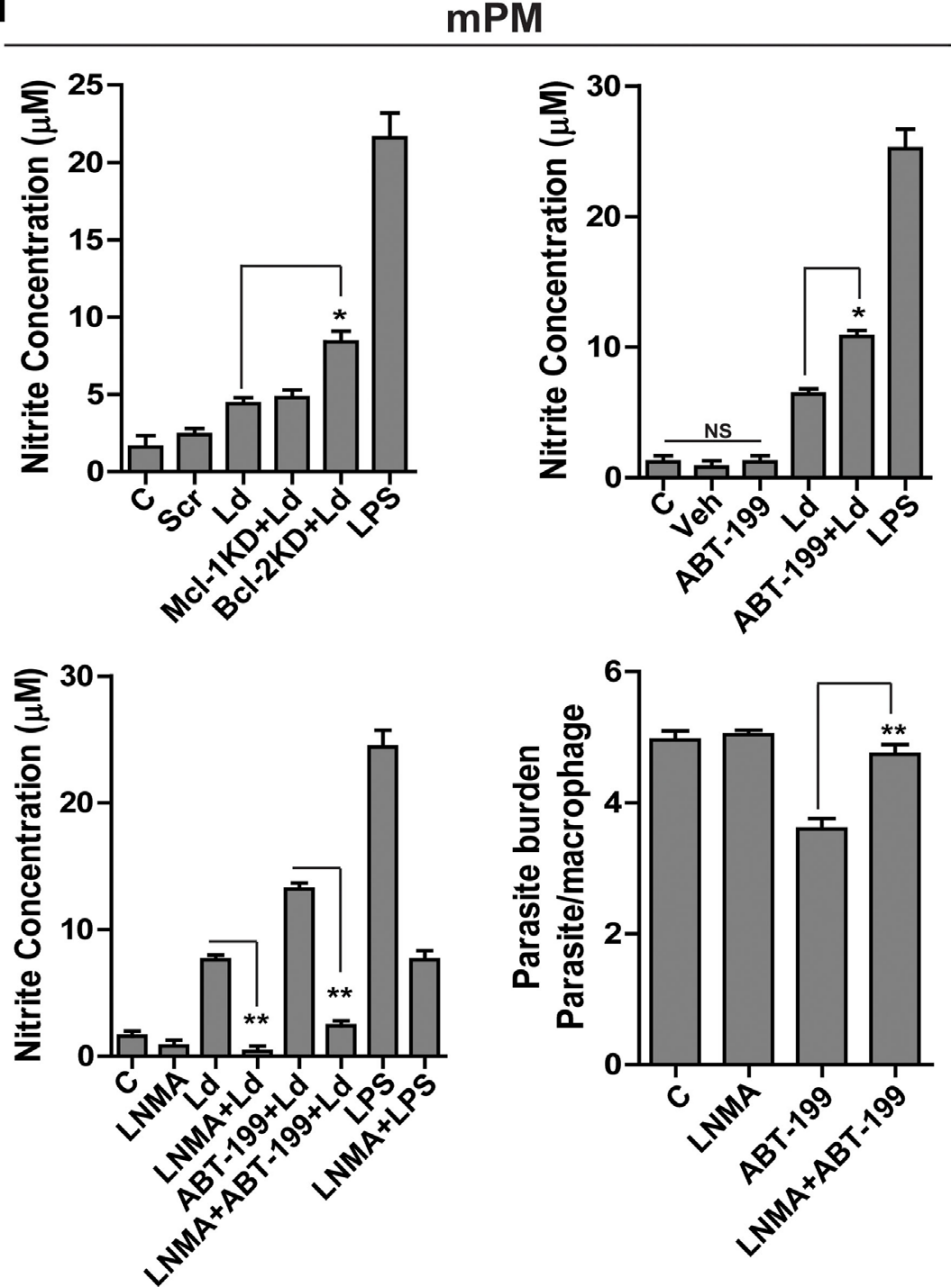
**Bcl-2 increase contributes to iNOS suppression**. **(A)** Western blot showing iNOS levels during control treatments and upon infection for 24 h. **(B)** iNOS levels at 12 and 24 h post infection. LPS has been taken as a positive control. **(C)** Blot showing higher iNOS expression at 12 h post-infection during Bcl-2 downregulation by siRNA to Bcl-2. Note no significant change after inhibition with siRNA to Mcl-1. Ld, *Leishmania donovani*; KD, knockdown; Scr, scrambled siRNA. **(D)** Western blot of THP1-MDM extracts at 12 h post-infection, showing higher levels of iNOS when Bcl-2 protein was inhibited by ABT-199/ABT-263. The bar graphs below the western blots in **(A–D)** show nitrite levels in soups obtained from corresponding experiments. Note the correspondence between iNOS expression and the nitrite levels in all the above experiments. **(E)** Nitrite concentrations at 12 h after treatment with Bcl-2 inhibitor ABT-199 and LNMA, iNOS inhibitor. Data are mean ± SEM (*n* = 3). *P ≤ 0.05; Mann–Whitney test. **(F)** Parasite burden 12 h postinfection in THP-1 MDM in the presence of Bcl-2 inhibitor ABT-199 and iNOS inhibitor, LNMA. Data are mean ± SEM (*n* = 3). ***P ≤ 0.001; Mann–Whitney test. **(G)** Photomicrograph of infected THP-1 MDM treated with LNMA and ABT-199. Note the loss of ABT-199-conferred reduction in parasite burden upon inhibition of iNOS activity by LNMA. The yellow arrows indicate intracellular parasites. **(H)** Representative Western blots from the lysates of hMDMs showing increased iNOS expression at 12 h upon Bcl-2 KD and in the presence of Bcl-2 inhibitors ABT-199/ABT-263. The bar graphs below represent corresponding nitrite levels. Ld, *Leishmania donovani*; KD, knockdown; Scr, scrambled siRNA. Data are mean ± SEM (*n* = 3). *P ≤ 0.05; Mann–Whitney test. **(I)** First three bar graphs show nitrite concentrations at 12 h after parasite infection of mPMs upon Bcl-2, Mcl-1 KD or treatments with Bcl-2 inhibitors, ABT-199/ABT-263 and upon inhibition of iNOS activity using LNMA treatment. The fourth bar graph shows increased parasite burden in LNMA-treated mPMs even in the presence of ABT-199. Ld, *Leishmania donovani*; KD, knockdown; Scr, scrambled siRNA; mPM, mouse peritoneal macrophage. Data are mean ± SEM (*n* = 3). *P ≤ 0.05, **P ≤ 0.01; Mann–Whitney test.

*Leishmania* parasites are sensitive to macrophage generated NO (13). Prior reports in other systems attribute NO regulation to Bcl-2 (16). With this background, we hypothesized an inhibitory role of excess Bcl-2 on NO production, as Bcl-2 knock down or its functional inhibition resulted in lower parasite burden (Figures 1D,E). Treatment with ABT-199 or the vehicle used for this inhibitor, DMSO did not have any effects on iNOS expression (Figure 2A). Infection of THP-1 MDMs resulted in a very low level induction of iNOS enzyme, which was also reflected in nitrite levels (Figures 2A,B). To look for a role of Bcl-2 protein in NO production during *L. donovani* infection, either Bcl-2 expression was downregulated using gene-specific siRNAs or Bcl-2 protein function was inhibited using ABT-199/ABT-263. A siRNA-mediated downregulation of Bcl-2, but not Mcl-1, resulted into a significant increase in iNOS levels (Figure 2C). Similarly, functional inhibition of Bcl-2 by ABT-199 or ABT-263 led to a significant induction of iNOS post infection (Figure 2D). Such interference with Bcl-2 expression or function also resulted in significantly higher nitrite production (Figures 2C,D), indicating higher iNOS activity. These data clearly demonstrated a negative effect of Bcl-2 on the NO generating machinery. Since higher iNOS level generally corresponds to increased NO production, to further confirm if increased iNOS expression, and consequently higher NO production was responsible for parasite killing in the presence of Bcl-2 inhibitors, cells were treated with an iNOS inhibitor, LNMA. Both NO production as well as parasite killing was abrogated upon iNOS inhibition (Figures 2E–G). Bcl-2 inhibition through ABT-199 or ABT-263 increased NO levels, but in the presence of LNMA this increase was not observed (Figure 2E). When parasite burden was estimated, a lower parasite burden was observed with both ABT-263 and ABT-199 treatment but this protection was significantly lost in the presence of LNMA (Figures 2F,G). Similar results were obtained with hMDMs (Figure 2H); the primary human macrophages demonstrated increased iNOS expression and nitrite levels on downregulation of Bcl-2 but not when Mcl-1 was lowered (Figure 2H).

To see if the parasite clearance seen upon Bcl-2 manipulation corresponded with NO levels in mPMs as observed in case of human macrophages, NO levels were quantified both during Bcl-2 downregulation and in the presence of Bcl-2 inhibitors (Figure 2I). Macrophages knocked down for Bcl-2 or treated with Bcl-2 inhibitors produced significantly more NO as compared to control cells. Both, NO production as well as increased parasite clearance achieved was abrogated upon iNOS inhibition (Figure 2I).

Collectively, the data from both human and mouse macrophages showed that parasite influenced NO production by modulating Bcl-2 expression. Functional inhibition of Bcl-2 protein augmented parasite killing owing to increased NO production.

### TLR-2 Is Required for Bcl-2 Increase

**Figure 3:**
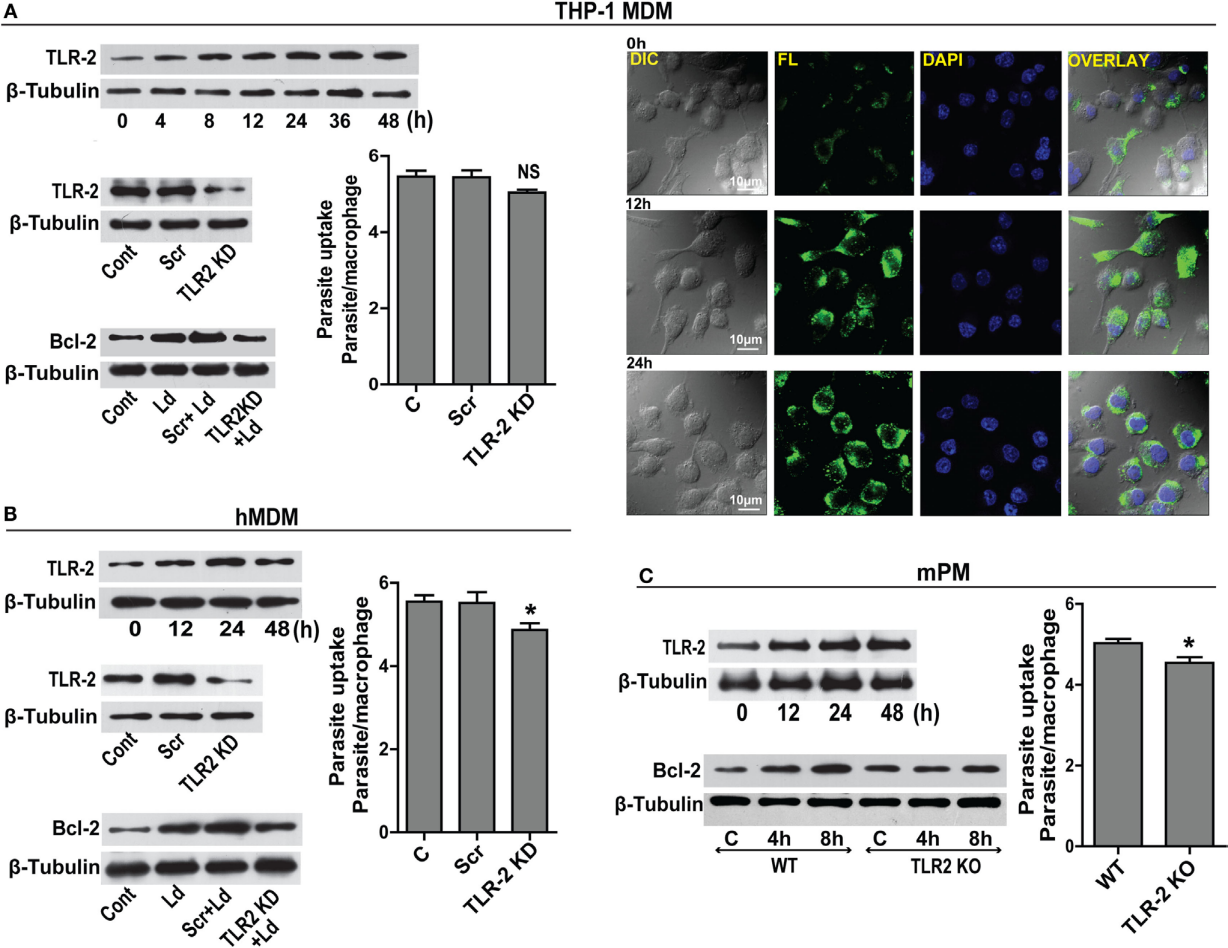
**TLR-2 expression increases upon infection**. **(A)** Western blot shows a time-dependent increase in TLR-2 expression by THP-1 MDMs after infection with *L. donovani* parasite. The blots below show siRNA-mediated KD of TLR-2 and reduced induction of Bcl-2 protein in TLR-2 KD macrophages. Blots are representative of three independent experiments. The bar graph alongside shows slightly reduced parasite uptake by TLR-2 KD THP-1 MDMs. Next placed are the immunofluorescence photomicrographs showing enhanced expression of macrophage TLR-2 receptors at 12 and 24 h post infection. **(B)** Blot shows increased TLR-2 expression by hMDMs upon *L. donovani* infection. The blot below shows infection-induced Bcl-2 expression was diminished in TLR-2 KD hMDMs. Ld, *Leishmania donovani*; KD, knockdown; Scr, scrambled siRNA. Blots are representative of three independent experiments. The adjacent bar graph shows a significant reduction in the parasite uptake by TLR-2 KD hMDMs. A minimum of 200 cells were counted. Data are mean ± SEM (*n* = 3). *P ≤ 0.05; Mann–Whitney test. **(C)** Blot shows a time-dependent increase in TLR-2 protein in mPM post infection. Unlike peritoneal macrophages from WT animals, no significant increase in the expression of Bcl-2 protein was observed in TLR-2 KO mPMs in response to infection. Blots in this figure are representative of at least 3–4 experiments. The adjacent bar graph shows that mPMs obtained from TLR-2 KO mice show significantly lesser uptake of parasites as compared to macrophages obtained from WT animals. The parasite uptake was measured by counting average number of internalized parasites per macrophage 3 h postinfection. WT, wild type; KO, knockout. Data are mean ± SEM (*n* = 3); *P ≤ 0.05; Mann–Whitney test.

Prior data suggest that *Leishmania* binds to macrophage surface through TLR-2 mediated by parasite surface lipophosphoglycans (20). TLR-2 is a pattern recognition receptor whose expression is reported to change during infection (21). A time-dependent upregulation of TLR-2 in THP-1 MDM post infection was observed (Figure 3A). This was confirmed by immunocytochemical staining for TLR-2 showing elevated immunoreactivity (Figure 3A). Based on prior knowledge of Bcl-2 regulation by TLR-2 in certain cell types, we explored the status of Bcl-2 under conditions of downregulated TLR-2. Significantly lesser induction of Bcl-2 was observed when TLR-2 was downregulated by siRNA transfection prior to infection (Figure 3A). As *Leishmania* parasite physically interacts with TLR-2 on the macrophage surface, downregulating TLR-2 expression may affect parasite internalization. To check if parasite uptake was affected in TLR-2 downregulated cells, unbound parasites were washed after 2 h of infection and a count of internalized parasites showed a minor reduction in the parasite uptake in TLR-2 siRNA-treated cells (Figure 3A). Despite being statistically insignificant, this minor reduction in the parasite uptake was consistently observed in multiple experiments. Similar to THP-1 MDM, hMDM showed an increase in TLR-2 expression post infection (Figure 3B), and TLR-2 downregulation prior to infection (Figure 3B) prevented the increase in Bcl-2 levels (Figure 3B). Unlike THP-1 MDM, statistically significant reduction in the parasite uptake in TLR-2 downregulated group was observed (*P* = 0.0161) (Figure 3B). The above observations were verified in cells from TLR-2 knockout mice. Wild-type mPM showed a gradual increase in TLR-2 expression post infection (Figure 3C) with an increase in Bcl-2 levels (Figure 3C). On the contrary, the peritoneal macrophages isolated from TLR-2 knockout mice of the same strain showed no significant increase in Bcl-2 levels post infection (Figure 3C). The mPMs derived from TLR-2 knockout mice also showed significantly reduced parasite uptake verifying that *Leishmania* parasites engaged TLR-2 for entry into cells (Figure 3C). Taken together, the data indicate involvement of TLR-2 in the regulation of Bcl-2 expression and also suggest a possible role of TLR-2 in parasite internalization.

### ERK Pathway Triggered in Response to *Leishmania* Infection Controls Bcl-2 Levels

**Figure 4:**
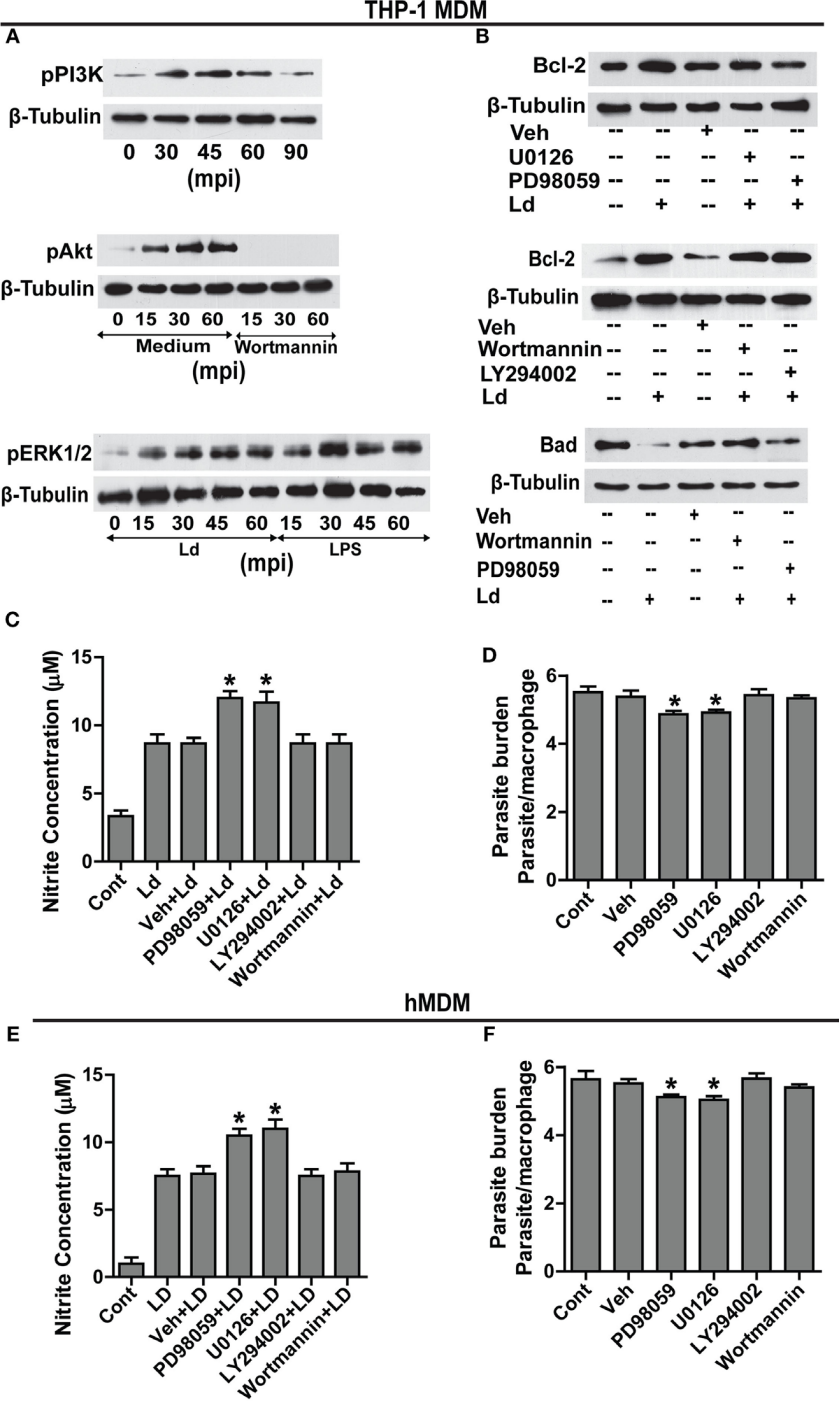
**MEK-MAPK/ERK but not PI3K-Akt activation regulates Bcl-2 increase in THP-1 MDM**. **(A)** Representative Western blots for pPI3K, pAkt, and pERK upon infection with *L. donovani*. PI3K is phosphorylated during early infection within 30–60 min. Note pretreatment with wortmannin, which is an inhibitor of PI3K phosphorylation, completely abrogates Akt phosphorylation. mpi, minutes post infection. **(B)** Blots showing effect of PI3K and ERK inhibitors on Bcl-2 induction during infection. The first blot shows significant decrease of infection-induced Bcl-2 on inhibition of ERK phosphorylation with U0126 and PD98059 (MEK1/2 inhibitors). The second blot in the figure shows no significant difference in the expression of Bcl-2 protein in the presence of PI3K inhibitors, wortmannin and LY294002 at 12 h postinfection. The third blot shows stabilization of the Bad protein in the presence of PI3K inhibitors showing PI3K mediated degradation of Bad protein during infection. Ld, *Leishmania donovani*; Veh, vehicle (DMSO). The blots in this figure are representative of three independent experiments. **(C)** Nitrite concentration measurements 12 h post infection in the presence of ERK or PI3K inhibitors. **(D)** Parasite burden in the presence of ERK or PI3K inhibitors. Note reduced parasite burden in the presence of ERK inhibitors. **(E,F)** show nitrite measurements and parasite burden respectively for hMDMs in the presence of ERK/PI3K inhibitors. A minimum of 200 cells was counted for estimating parasite burden. Data are mean ± SEM (*n* = 3); *P ≤ 0.05; Mann–Whitney test.

Searching for downstream effectors of TLR-2, changes in pPI3K, pAkt, and pERK1/2 were determined. Parasite challenge caused a time-dependent increase in phosphorylation of PI3K, Akt, and ERK1/2 (Figure 4A). Therefore, to determine the relationship between pPI3K, pAkt, pERK1/2, and Bcl-2 induction during infection, specific pharmacological inhibitors were used to block the kinase activities of the proteins leading to phosphorylation of PI3K, Akt, and ERK1/2. Inhibition of ERK 1/2 phosphorylation with U0126 resulted in an observable reduction in the infection-induced Bcl-2 increase (Figure 4B). Both PD98059 and U0126, MAPK/ERK kinase inhibitors significantly prevented Bcl-2 induction (Figure 4B) suggesting regulation of Bcl-2 through the MEK/ERK pathway. The inability of wortmannin and LY294002, the PI3K inhibitors to prevent the increase in Bcl-2 post infection (Figure 4B) ruled out the involvement of the PI3K pathway in Bcl-2 changes. NO levels were found to be significantly increased in the presence of ERK inhibitors U0126 and PD98059 during parasite invasion (Figure 4C) suggesting higher iNOS activity, whereas, in the presence of wortmannin and LY294002, nitrite levels remained at par with only parasite-infected cells (Figure 4C). In consonance with the above data, presence of both U0126 and PD98059 showed reduced infection (Figure 4D) suggesting higher NO levels to be effective against the parasite, whereas, the presence of wortmannin did not interfere with parasite burden (Figure 4C). The hMDMs also produced significantly higher NO in the presence of ERK inhibitors (Figure 4E) and showed significantly reduced parasite burden (Figure 4F). In summary, this part of the data showed participation of MEK/ERK signaling pathway in *L. donovani*-induced increases in Bcl-2 levels, lowering of NO production and higher infection rates. Clearly, MEK/ERK acted as the downstream effectors of TLR-2 after infection with the *L. donovani* parasite leading to Bcl-2 increase favoring parasite survival.

### IL-13 Regulates Bcl-2 through STAT-3

**Figure 5:**
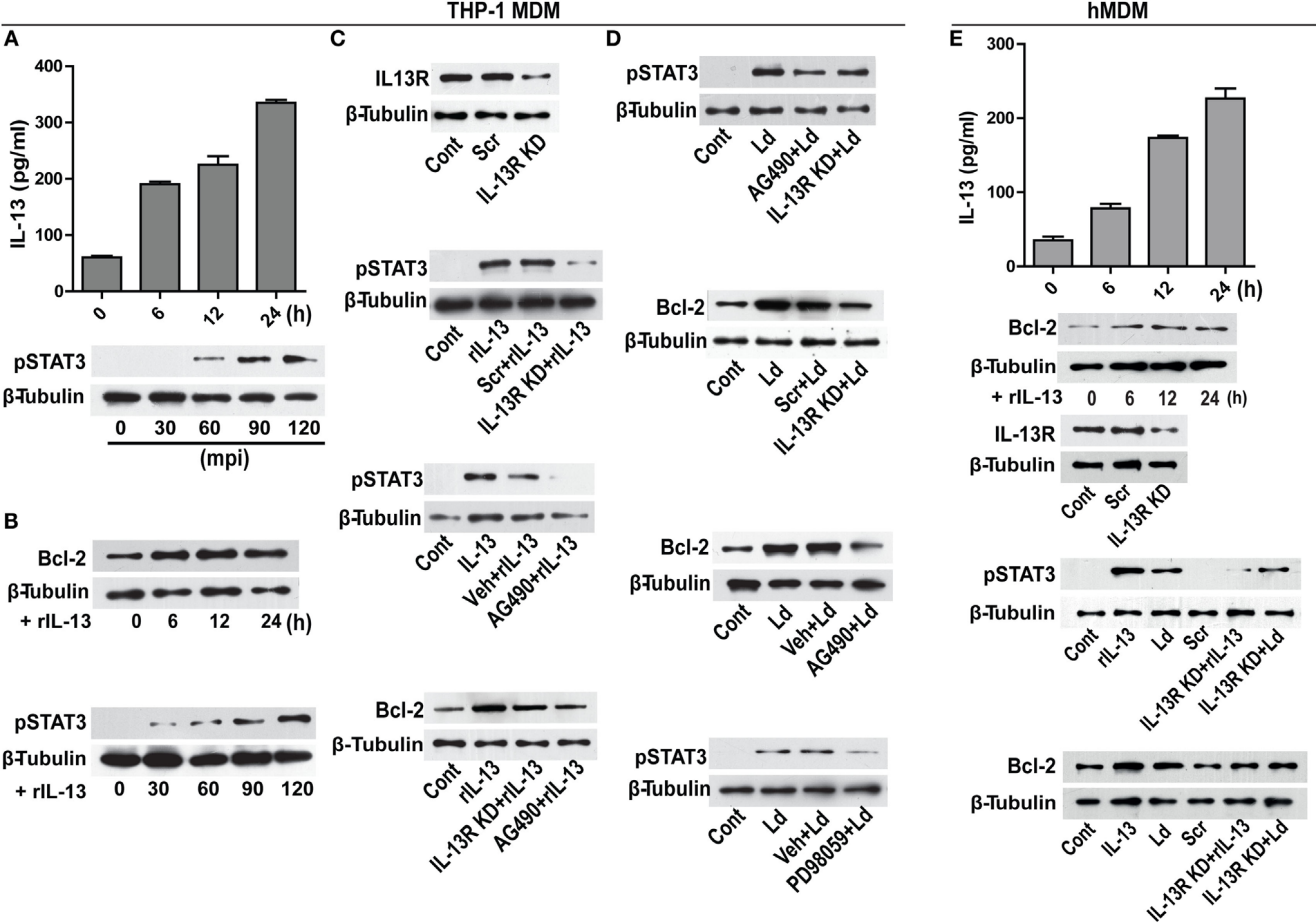
**IL-13 regulates Bcl-2 expression through STAT-3 activation**. **(A)** A time-dependent increase in IL-13 secretion post infection *in vitro*. Data are mean ± SEM (*n* = 3), **P < 0.01, Mann–Whitney test. Western blot shown below demonstrates early phosphorylation of STAT-3 in response to infection. mpi, minutes post infection. **(B)** Blots showing increase in Bcl-2 expression and STAT-3 phosphorylation at different time points *in vitro* upon treatment with rIL-13 (1 ng/ml). **(C)** Western blot showing siRNA mediated downregulation of IL-13 receptors. Blots shown below demonstrate a reduction in STAT-3 phosphorylation in IL-13R downregulated cells or in the presence of AG490, a JAK2 inhibitor. Blot further below shows a significant decrease in rIL-13-induced Bcl-2 expression both in IL-13R knockdown cells as well as in cells treated with AG490. Cont, no treatment. Scr, scrambled siRNA; KD, knockdown; rIL-13, recombinant IL-13. **(D)** Blots show reduced phosphorylation of STAT-3 when expression of IL-13R was reduced (as compared to scrambled siRNA treated cells) as well as after pretreatment with AG490 at 90 min post infection. Blots below show Bcl-2 levels in infected cells with downregulated IL-13R or JAK-2 inhibition. Blot immediately below shows reduction in phosphorylated STAT-3 upon PD98059 treatment. LD, *Leishmania donovani*. Cont, no infection; Veh, vehicle (DMSO). Blots are representative of at least three experiments. **(E)** hMDMs show a time-dependent increase in secretion of IL-13 upon infection *in vitro*. Western blot immediately below shows increase in Bcl-2 expression upon treatment with rIL-13 (1 ng/ml). Blot below shows IL-13 receptor downregulation upon IL-13 receptor siRNA treatment. Blots further below show that both rIL-13/infection-induced STAT-3 phosphorylation as well as Bcl-2 induction are significantly reduced upon IL-13R KD or JAK-2 inhibition. LD, *Leishmania donovani*; KD, knockdown; rIL-13, recombinant IL-13; IL-13R KD, IL-13 receptor knockdown.

Modulation of cytokines is an important event during infection. From our *in vitro* studies, it was observed that challenge with *L. donovani* parasite caused a time-dependent increase in IL-13 levels (Figure 5A). A report involving patient data has shown increased level of IL-13 in the VL sera (22) that has been also confirmed in our study with human VL subjects (unpublished data). Therefore, our *in vitro* observations were similar to the previously reported patient data. IL-13 is responsible for inducing a host of different functions on different kind of cell types including macrophages (23). As it is known that Bcl-2 levels are regulated by IL-13 *via* STAT-3 in certain cell types (24), we sought to explore if Bcl-2 was responding to IL-13 signals. THP-1 MDMs were exposed to recombinant IL-13 (rIL-13) and a time-dependent increase in Bcl-2 levels and increase in the phosphorylation of STAT-3 was recorded (Figure 5B). STAT-3 phosphorylation is a downstream event of IL-13 exposure, as is known in other cell types of the immune lineage. To further establish the role of IL-13 in Bcl-2 induction, IL-13 signaling was perturbed at different levels: at the levels of IL-13 receptor (IL-13R) and JAK-2, which is the upstream kinase responsible for phosphorylating STAT-3. THP-1 MDMs with IL-13R downregulation using siRNAs to IL-13R or pretreated with AG490, an inhibitor of JAK-2 displayed reduced phosphorylation of STAT-3 (Figure 5C) upon treatment with rIL-13. These conditions also prevented Bcl-2 increases during exposure to rIL-13 (Figure 5C). Importantly, infection-induced STAT-3 phosphorylation and Bcl-2 increase declined when cells were challenged with parasites in IL-13R downregulated conditions or in the presence of AG490 (Figure 5D). Reduced phosphorylation of STAT-3 was also observed in the presence of PD98059, an ERK inhibitor (Figure 5D), indicating an involvement of pERK 1/2 in STAT-3 phosphorylation in addition to IL-13-JAK-2 axis during infection. This finding also suggested convergence of TLR-2-ERK and IL-13-JAK-2 axes at the level of STAT-3. In all, the data suggested that infection-induced induction of Bcl-2 was predominantly mediated through STAT-3.

Infection of hMDMs with *L. donovani* parasites generated a similar response like that of THP-1 MDMs by inducing a time-dependent increase in IL-13 (Figure 5E). The rIL-13 administration to naive hMDMs induced a Bcl-2 increase similar to THP-1 MDMs (Figure 5E). Under IL-13R downregulated conditions, both rIL-13 and the *Leishmania* parasites were unable to induce significant STAT-3 phosphorylation and Bcl-2 increase (Figure 5E) in hMDMs. In summary, in both THP-1 MDMs and hMDMs, IL-13 contributed to Bcl-2 induction *via* STAT-3 phosphorylation.

### *In Vivo* Inhibition of Bcl-2 Abrogates Infection

**Figure 6:**
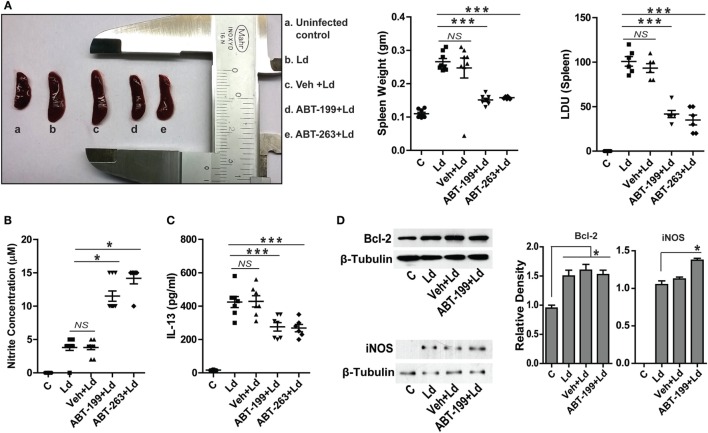
**ABT-199 treatment reduces parasite burden in mice**. **(A)** *L. donovani* infection-induced splenomegaly in mice (b) which shows significant reduction in ABT-199 (d) and ABT-263 (e) treated group. Scatter plots show spleen weight and splenic parasite burden in terms of Donovan units. Note the reduction in total splenic weight as well as parasite burden in ABT-199- and ABT-263-treated group. LDU, Leishman Donovan units; Ld, *Leishmania donovani*; Veh, vehicle (DMSO). **(B)** Griess’ assay shows a significant increase in the serum nitrite levels in ABT-199 as well as ABT-263 treated mice as compared to only infection or vehicle treated controls, with negligible concentrations of serum nitrite. **(C)** IL-13 ELISA shows elevated levels of IL-13 cytokine in the serum of mice infected with *L. donovani*. Note that ABT-199- and ABT-263-treated mice show lower levels of IL-13. Vehicle controls show IL-13 levels comparable to only infection group. Data in **(A–C)** are mean ± SEM (*n* = 6–8); *P ≤ 0.05, ***P < 0.001; Mann–Whitney test. **(D)** Western blots from crude splenic lysates show increased Bcl-2 expression in all infected groups. ABT-199 treatment had no effect on the Bcl-2 expression. On the other hand ABT-199-treated group showed increased iNOS expression. Adjacent bar graphs show the densitometric plots for the Bcl-2 and iNOS blots respectively. Ld, *Leishmania donovani*; Veh, vehicle (DMSO). *P ≤ 0.05; Mann–Whitney test.

It was important to test the occurrence and relevance of the *in vitro* findings in an animal model of VL. To test the hypothetical role of Bcl-2 inhibition on the disease outcome *in vivo, L. donovani* infection was established in BALB/c mice and the role of Bcl-2 small molecule inhibitors, ABT-199 and ABT-263, was investigated. It should be noted that the inoculums used to infect mice were derived directly from the primary cultures of the parasites in the splenic aspirate of a VL patient. Splenomegaly is a common feature of VL where the size of the spleen is in general an indication of the parasitic burden and, therefore, the active disease. Mice treated with the Bcl-2 small molecule inhibitors showed significantly lesser spleen size and weight as compared to that from mice without the inhibitor treatments (Figure 6A). In support of the above data, the number of infected macrophages per spleen was observed to be significantly higher in control and only vehicle-treated groups as compared to ABT-199- and ABT-263-treated animals (Figure 6A), suggesting reduction in the number of infected macrophages in low Bcl-2 conditions. The *in vitro* observation of increased cellular NO production when Bcl-2 was inhibited was confirmed *in vivo* where ABT-199- and ABT-263-treated mice showed significantly higher serum nitrite levels as compared to vehicle-treated controls (Figure 6B). Infection-induced increase in IL-13 observed *in vitro* was also visible in the *in vivo* model where serum IL-13 levels were consistently higher in the infected animals (Figure 6C). On the other hand, ABT-199- and ABT-263-treated groups showed significantly lesser serum IL-13 (Figure 6C). When lysates from mouse spleen were checked for Bcl-2 protein, infected mice from all groups showed elevated Bcl-2 levels as compared to the uninfected control (Figure 6D). ABT-199 treatment apparently had no effect on Bcl-2 expression *in vivo*. While lysates from uninfected control mice showed no expression of the iNOS protein, barely detectable iNOS levels were visualized from *L. donovani*-infected group (Figure 6D). Interestingly, higher iNOS levels were found in the lysates from ABT-199-treated group mice (Figure 6D), possibly accounting for higher nitrite levels in the sera of ABT-199 treated. The *in vivo* data showing decreased parasite burden when infected mice were treated with Bcl-2 inhibitors clearly indicated an antileishmanial effect of the drugs. The confirmation of higher nitrite levels *in vivo* corroborated the data obtained at the cellular level suggesting NO-mediated killing of the parasites. Taken together, the data from *in vitro* experiments were reproducible in the animal model.

### Human VL Subjects Show Elevated Bcl-2 Levels and Deficient iNOS Expression

**Figure 7:**
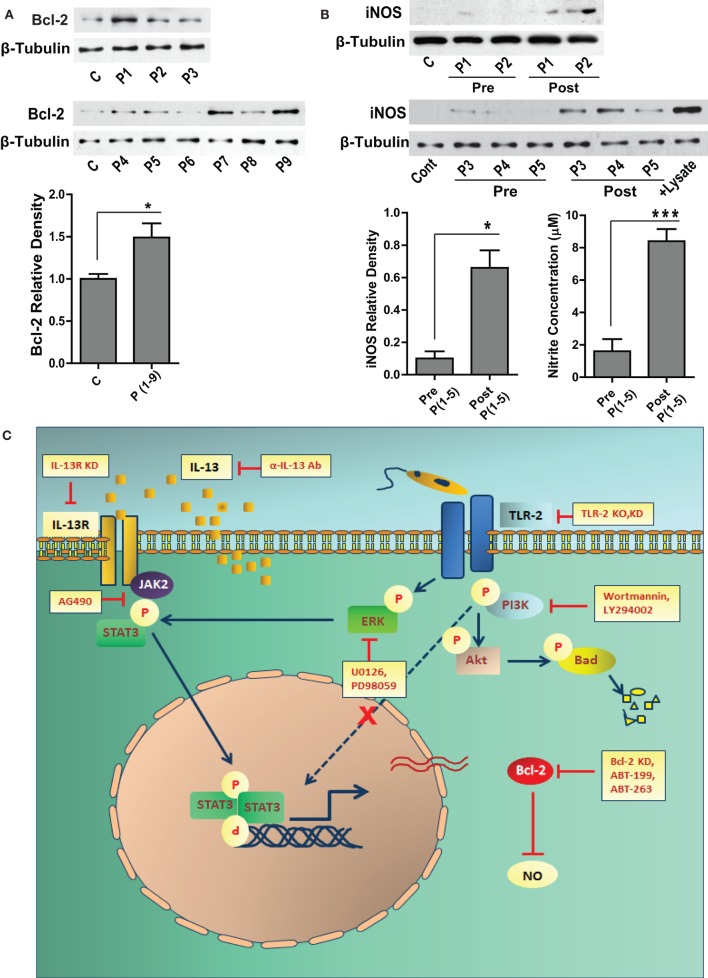
**Bcl-2 and iNOS protein levels in the human VL subjects**. **(A)** The western blot shows Bcl-2 expression in the PBMCs obtained from the blood of human VL patients. The adjacent bar graph shows densitometric average of the patient (P1-P9) Bcl-2 levels as compared to healthy controls. *P ≤ 0.05; Mann–Whitney test. **(B)** Blots show iNOS protein levels in the PBMCs of VL patients (P1–P5), pre and post treatment. The adjacent plot shows densitometric comparison of iNOS levels pre and post treatment. The next bar graph shows nitrite measurements in the serum of the same patients, pre and post treatment. *P ≤ 0.05; Wilcoxon signed-rank test. **(C)** Schematic showing the involvement of signaling pathways during *Leishmania* infection.

Sera from VL patients show significantly elevated levels of IL-13 (22), which may result into its downstream biological effects in the cells of monocyte lineage. Our *in vitro* data showed increased expression of Bcl-2 in response to IL-13, therefore to look at the status of Bcl-2 in the human subjects, PBMCs were isolated and Bcl-2 expression was checked in the lysates. Six out of nine VL patients presented with increased Bcl-2 expression as compared to the healthy controls (Figure 7A). Based on our *in vitro* data on human macrophages, increased Bcl-2 expression corresponded to deficient NO response. Therefore, it was important to look at the same in the VL patients. PBMCs from VL patients expressed barely detectable levels of the iNOS protein, which was also reflected in the nitrite levels in the corresponding sera (Figure 7B). When paired patients samples were checked for iNOS, a substantial increase in the expression was observed posttreatment which was again reflected in the level of serum nitrites (Figure 7B).

## Discussion

In almost every instance of a pathogenic invasion, the most conspicuous motive behind manipulation of the Bcl-2 family members is to establish a safer niche where the pathogen may reside and multiply itself to bring about a state of an established infection. In this context, even a highly virulent pathogen is continuously under the hazard of being non-selectively killed due to induction of apoptosis in the infected host cell. Therefore, apoptosis represents a very primitive and highly evolved innate immune mechanism and a successful pathogen may continuously need to be able to manipulate it. Even though a substantial amount of literature exists on the various signaling molecules manipulated by the *Leishmania* parasite (25), there are no reports on how the pro and antiapoptotic molecules of the Bcl-2 family behave in response to *L. donovani* infection. This is important from two points of view; first, because Bcl-2 family of proteins control a variety of cellular functions and therefore, the possibility of them being manipulated by the parasite is high and, second, Bcl-2 is already an established drug target in diseases like cancer where it is upregulated (26, 27), and therefore, if functionally important could also be considered as a drug target in leishmaniasis. In this report, we present evidence of a novel role of Bcl-2 protein in the sustenance of *L. donovani* infection and suggest that it could be a molecule for evaluation as a potential drug target.

Our observations showing increased expression of Bcl-2 and Mcl-1 proteins during *L. donovani* infection are in consonance with reports of enhancement in the levels of antiapoptotic members of the Bcl-2 family during viral and bacterial infections like HIV-1 (28) and *Mycobacterium tuberculosis* (11). Unlike the mycobacterial system, where creating a deficiency of the antiapoptotic Bcl-2 proteins resulted in accelerated bacterial clearance due to enhanced apoptosis of the infected host cells, host macrophages harboring *Leishmania* parasites did not readily undergo apoptosis even in a background deficient for Bcl-2 or Mcl-1. However, macrophages consistently showed significantly reduced number of parasites under a Bcl-2-deficient background. The experiments showing reduction in the parasite numbers during siRNA-mediated downregulation of host Bcl-2 but not Mcl-1 suggested specific involvement of Bcl-2 in the parasite survival through mechanism/s independent of apoptosis. Although pathogens like *Mycobacterium tuberculosis* (29), *Trichomonas vaginalis* (30), *Streptococcus pneumoniae* (31), and hepatitis C virus (32) use Mcl-1 to their advantage, Mcl-1 levels in our studies did not appear to have significant effect on *Leishmania* survival *per se*. However, *Leishmania*-infected macrophages knocked-down for Mcl-1 exhibited statistically higher mortality at 72 h postinfection. In this context, increased Mcl-1 levels, in addition to Bcl-2 may also confer a survival advantage to the infected macrophages. Generation of microbicidal molecules, like NO or reactive oxygen species, by the host macrophages poses a major challenge to the invading parasite (33). Our results showed significantly increased iNOS activity during infection, when either Bcl-2 expression or function was inhibited, suggesting an inhibitory role of Bcl-2 on NO production. To our knowledge, this is the first report where a very close involvement of host Bcl-2 in infection related events other than regulation of host-cell apoptosis has been demonstrated during a parasitic infection. The reversal of parasite elimination by LNMA, an iNOS inhibitor, even in the presence of Bcl-2 inhibitors suggested a crucial role of iNOS activity in parasite killing. Defective NO response after infection leads to resistance to antimonial drugs, which form the first line of therapy against leishmaniasis (34) indicating importance of this reactive species in the antimicrobial arsenal of the host cells. In addition to NO, reactive oxygen intermediates (ROIs) do contribute to parasite clearance (35); however, under these circumstances NO seems to be critical for *Leishmania* killing. The role of Bcl-2 in regulating microbicidal activities in the macrophages in response to *L. donovani* infection shown in this study is a novel observation, suggesting a potential use of Bcl-2 manipulation for interfering with parasite survival.

Having established an important role of Bcl-2 in the regulation of NO, two things became important in the purview of our study: to understand the mechanism/s which led to Bcl-2 induction in the first place, and, second, how the increased Bcl-2 levels may affect iNOS expression or activity. To address the first question, we took some insight from the cancer models, where Bcl-2 overexpression is the etiological factor (36). Bcl-2 induction in some of these transformed cells has been attributed to IL-13-mediated activation of STAT-3 (37). STAT-3 upon activation-induced dimerization translocates to the nucleus where it may bind to the Bcl-2 promoter with high affinity and cause robust Bcl-2 induction (37). Cytokine profiling of *Leishmania*-infected macrophages showed increased expression of IL-13 in the supernatant, entailing possible involvement of IL-13 signaling as a cause of Bcl-2 induction. Our results indeed supported this hypothesis where infection-induced expression of Bcl-2 was shown to be majorly regulated by the IL-13-JAK2-STAT-3 axis. Manipulations both at the level of IL-13R as well as JAK-2 resulted in significant abrogation of Bcl-2 induction. IL-13 is a pleiotropic cytokine produced by Th-2 cells, with anti-inflammatory properties having a major role in allergic inflammation; however, the effects of IL-13 appear to be context and model specific. In a mouse model, IL-13 was described as a susceptibility factor during *L. major* infection (38). An earlier report has shown presence of IL-13 in the sera of VL patients (22) but it is not known if this cytokine plays any role in the etiology of VL. By demonstrating the role of IL-13 in the regulation of Bcl-2 expression, our studies demonstrate a previously unexplored pro-parasitic action of this cytokine and propose a role for this cytokine in the pathogenesis of VL.

Toll-like receptor-2 is a pattern recognition receptor associated with recognition of LPG on the surface of the *Leishmania* parasite (20). Engagement of TLR-2 may lead to activation of myriads of signaling cascades, including PI3K (39) and ERK (40). Either of these kinases is known to induce Bcl-2 in mammalian cells (41). We found activation of both of these molecules at early stages during infection; however, only the latter was found to mediate *Leishmania*-induced Bcl-2 expression. Interestingly, inhibition of ERK phosphorylation also resulted in partial inhibition of STAT-3 activation, showing convergence of IL-13-JAK-2 and TLR-2-ERK pathways at the level of STAT-3 to regulate Bcl-2 expression. The schematic presented in Figure 7C summarizes these findings. Our *in vitro* data therefore suggested role of cascades downstream of TLR-2 in the regulation of Bcl-2 family members during *Leishmania* infection. TLR-2 on the macrophage surface also appeared to facilitate parasite internalization, at least in primary macrophages. These results suggest a defining role of the early interaction between the parasite and the TLR-2 molecules on the host-cell surface.

To establish these finding *in vivo*, we utilized a mouse model of VL. Mouse and hamster are the two established animal models of VL (42). Hamsters are characterized by a generalized deficiency in their capacity to generate NO, and to a certain extent ROIs (43). Not only is it easier to establish *L. donovani* infection in hamsters, they also show a relatively rapid progression to the state of a full blown VL phenotype. This inherent deficiency to mount a detectable NO response may account for such rapid progression of the active disease. Owing to the reason that our *in vitro* data showed a strong implication of the NO response, choice of mouse as our animal model was obvious. The results coming from animal experiments showed good agreement with our *in vitro* data, where higher expression of Bcl-2 protein was observed in the splenic tissue of infected mice and treatment with Bcl-2 inhibitors showed significantly reduced splenic size/weight as well as parasite burden. Treatment with Bcl-2 inhibitors also resulted into a better NO response by the infected mice. Infected mice consistently showed elevated levels of IL-13 in their sera, which dramatically reduced upon treatment with the Bcl-2 inhibitors and was in close correspondence with the parasitic burden. These findings were *in vivo* validation of the earlier observations, on the basis of which we had hypothesized a functional role of infection-induced Bcl-2 in the pathogenesis of VL and had proposed use of Bcl-2 inhibitors as a therapeutic intervention. Taking this work one step further, we were able to see the actual Bcl-2 and NO levels in the human VL patients, where PBMCs from six out of nine patients presented with higher Bcl-2 expression. When iNOS protein expression was observed in the paired samples, a fair increase in iNOS expression was observed post treatment. Patients prior to treatment with antileishmanial drugs showed no detectable expression of the iNOS protein, indicating complete suppression of a functional NO response during VL. The differences in the serum nitrite levels were even starker as we observed significantly higher levels of NO in the patient sera post treatment.

Collectively, this study provides a strong evidence of involvement of the host Bcl-2 protein in the sustenance of *L. donovani* infection and defines a novel mechanism through which host-derived IL-13 mediates a pro-parasitic role. Diminution of infection on Bcl-2 inhibition provides an opportunity for using Bcl-2 as an antileishmanial target.

## Author Contributions

CS and RP conceived and designed the experiments. RP, SM, and S. Sharma performed the experiments. CS and RP analyzed the data. CS, S. Sundar, and RG contributed reagents. CS and RP wrote the paper.

## Conflict of Interest Statement

The authors declare that the research was conducted in the absence of any commercial or financial relationships that could be construed as a potential conflict of interest.

## Supplementary Material

Figure S1 ***Leishmania donovani* infection of different macrophage cell types**. **(A)** Confocal photomicrographs of THP-1MDMs, hMDMs and mPMs infected with *L. donovani* at an MOI of 1 macrophage:10 parasites. The Bar graph shows average infection counts at MOIs of 1:10 and 1:20 respectively in the three aforementioned cell types *in vitro*/*ex vivo*. The infection ratios shown here are averages obtained from counting a minimum of 200 cells from at least 15 randomly selected fields. **(B)** Plot shows % apoptosis in the THP-1 MDMs upon infection at different time points. The *y*-axis shows percentage of Annexin positive cells. Cells were either left untreated or were knocked down for Bcl-2 or Mcl-1. Cells transfected with scrambled siRNAs were taken as a control.
