# Supplementary figures and images for: Leishmania donovani-Induced Increase in Macrophage Bcl-2 Favors Parasite Survival
Source: Front Immunol. 2016 Oct 25;7:456. doi: 10.3389/fimmu.2016.00456 (PMC5078497; doi:10.3389/fimmu.2016.00456)

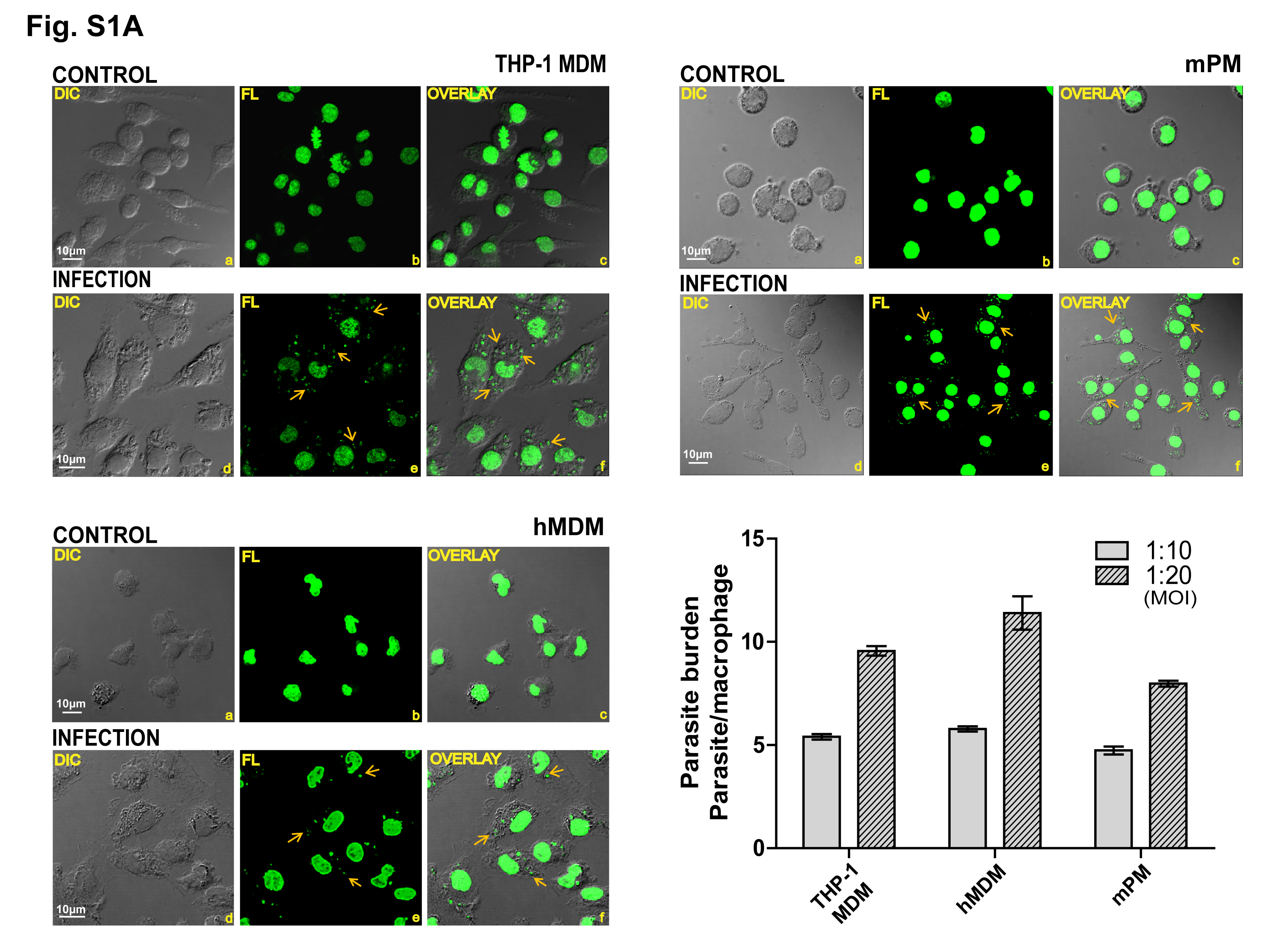

Supplement: Supplementary file 1 [file Image_1.TIF]

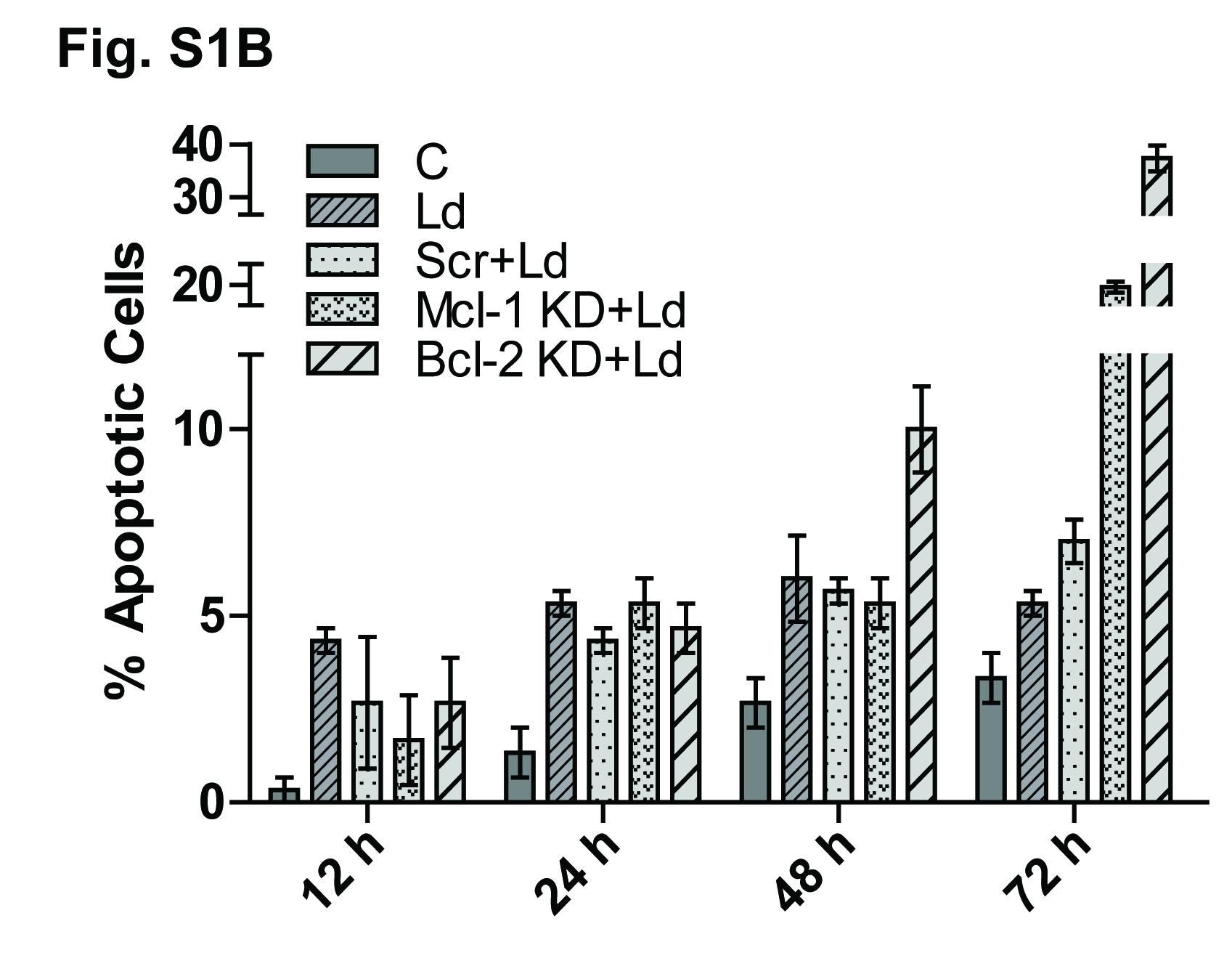

Supplement: Figure S1 — Leishmania donovani infection of different macrophage cell types. (A) Confocal photomicrographs of THP-1MDMs, hMDMs and mPMs infected with L. donovani at an MOI of 1 macrophage:10 parasites. The Bar graph shows average infection counts at MOIs of 1:10 and 1:20 respectively in the three aforementioned cell types in vitro/ex vivo. The infection ratios shown here are averages obtained from counting a minimum of 200 cells from at least 15 randomly selected fields. (B) Plot shows % apoptosis in the THP-1 MDMs upon infection at different time points. The y-axis shows percentage of Annexin positive cells. Cells were either left untreated or were knocked down for Bcl-2 or Mcl-1. Cells transfected with scrambled siRNAs were taken as a control. [file Image_2.TIF]
